# Analytical Determination of the Brake Temperature Mode during Repetitive Short-Term Braking

**DOI:** 10.3390/ma14081912

**Published:** 2021-04-11

**Authors:** Aleksander Yevtushenko, Katarzyna Topczewska, Michal Kuciej

**Affiliations:** Faculty of Mechanical Engineering, Bialystok University of Technology (BUT), 45C Wiejska Street, 15-351 Białystok, Poland; a.yevtushenko@pb.edu.pl (A.Y.); k.topczewska@pb.edu.pl (K.T.)

**Keywords:** repetitive short-term braking, frictional heating, temperature, thermal sensitivity of materials, friction coefficient

## Abstract

An algorithm to determine the maximum temperature of brake systems during repetitive short-term (RST) braking mode has been proposed. For this purpose, the intermittent mode of braking was given in the form of a few cyclic stages consisting of subsequent braking and acceleration processes. Based on the Chichinadze’s hypothesis of temperature summation, the evolutions of the maximum temperature during each cycle were calculated as the sum of the mean temperature on the nominal contact surface of the friction pair elements and temperature attained on the real contact areas (flash temperature). In order to find the first component, the analytical solution to the one-dimensional thermal problem of friction for two semi-spaces taking into account frictional heat generation was adapted. To find the flash temperature, the solution to the problem for the semi-infinite rod sliding with variable velocity against a smooth surface was used. In both solutions, the temperature-dependent coefficient of friction and thermal sensitivity of materials were taken into account. Numerical calculations were carried out for disc and drum brake systems. The obtained temporal variations of sliding velocity, friction power and temperature were investigated on each stage of braking. It was found that the obtained results agree well with the corresponding data established by finite element and finite-difference methods.

## 1. Introduction

Repetitive short-term mode of braking (RST) is a sequential performance of a certain number of cycles consisting of two stages: braking (heating) and acceleration (cooling) [1]. In analytical and numerical models, the main characteristics to describe the braking process (temporal profiles of velocity and friction power, time to standstill, maximum temperature, etc.) are found from solutions to the following problems [2,3]:

1. initial value problem for vehicle motion;
2. boundary-value problem of heat conduction, taking into account frictional heat generation (the so-called thermal problem of friction).

The schemes of the characteristics’ determination, consisting of subsequent solutions to the above-mentioned problems, are the so-called uncoupled models. In this kind of scheme, to determine the sliding velocity profile, time to stop and resulting evolution of specific friction power, first, the initial value problem for the equation of motion of braking system has to be considered. After finding the specific friction power in this manner, its temporal profile is adapted to one of the boundary conditions during formulation of the boundary-value problem of heat conduction. The equality of the specific friction power and the sum of heat flux intensities, directed inside each element of the friction pair perpendicular to the contact surface, is required in this condition. The disadvantage of such an approach is that, when obtaining solutions, a constant, usually averaged with the braking time, the value of the friction coefficient is used. In consequence, the calculations do not take into account the mutual influence of sliding velocity and temperature.

Numerical uncoupled models of braking in the RST mode, based on the finite element method (FEM), were developed for automotive [4,5,6,7] and railway [8] brakes. The influence of convective cooling of free surfaces on temperature [4] and thermal stresses were studied in [5,6,7]. A comparative analysis of volumetric and mean temperature on the friction surface of the disc brake of the diesel multiple unit, obtained by means of: (a) numerical solution to the spatial thermal problem of friction for a disc-pad system, (b) analytical solution to the corresponding one-dimensional problem [9], and (c) experimental data using thermocouples [10] was carried out in [8]. To predict temperature, a computer model of an automotive disc brake operation during short-term multiple brakings was developed [11]. A theoretical and experimental methodology for determining the heat transfer coefficient during the RST braking mode has been proposed [12].

However, if for a selected friction pair the experimental data of frictional thermal stability are known, then the temperature calculations can be made according to the coupled models [13,14]. In this kind of model, the above-mentioned problems are related to the temperature-dependent coefficient of friction, and their solutions at each time step are obtained simultaneously. The implementation of the coupled calculation scheme allows to take into account the interdependence of sliding velocity and temperature during each stage of the RST braking mode. Since both of the above-mentioned problems in the coupled models are non-linear, their solutions were obtained numerically, using FEM [15] or the finite differences method (FDM) [16].

The use of numerical methods allows to take into account the actual dimensions of the braking system, including the contact area of the pad and disc, as well as the cooling of free surfaces, thermal sensitivity of materials, etc. On the other hand, the use of numerical methods requires researcher to have advanced skills when selecting the space–time grid, controlling the stability of calculations, performing verification of the obtained results with appropriate experimental data, etc. Therefore, analytical methods of solving thermal problems of friction during braking are still being investigated, simultaneously with numerical and experimental methods. These are mainly linear boundary value problems of heat conduction [17]. The exact obtained solutions to such problems during single braking cases are in the form of closed engineering formulas and allow for instant assessment of the temperature during braking under light conditions with sufficient accuracy; when the volumetric and the average temperatures of the friction surface do not exceed 100 °C and 200 °C, respectively [18,19,20]. Under heavy and medium braking conditions, when the volume temperature reaches values above 250 °C, the friction coefficient and properties of the friction pair materials change significantly due to the influence of temperature; therefore, numerical methods are used [21,22]. An attempt was made to use the exact solutions of linear thermal problems of friction to determine temperature during single braking of a system made of thermally sensitive friction materials [23,24,25].

It should be noted that, in the sense of methodology, the results contained in the monograph by A.V. Chichinadze et al. [9] and in [16] are closest to our study. The algorithm for determining the maximum temperature of a braking system in [9] is based on an analytical solution to the thermal problem of friction during single braking for two layers. This solution was obtained with two very important simplifying assumptions. The first one is the presumption that the temperature of any point on the axis, perpendicular to the friction surface, is directly proportional to the braking time. The second assumption is that the temperature increase, in this case, is equal to the increase in the mean volumetric temperature of the system. With such simplifications, this solution can be defined as approximate in an analytical form. A detailed comparative analysis of the temperature fields during single braking, obtained with the use of an approximate solution [9] and the exact solution, was carried out in [18]. The numerical FDM solution to the thermal problem of friction for the RST operating mode of a disc brake was obtained in [16]. This solution also belongs to the class of approximate solutions; whereas the main purpose of this study was to obtain exact engineering formulae to this problem and the development, on the basis of them, of a corresponding calculation scheme to find the maximum temperature in a braking system operating in RST mode.

## 2. Statement of the Problem

**Figure 1:**
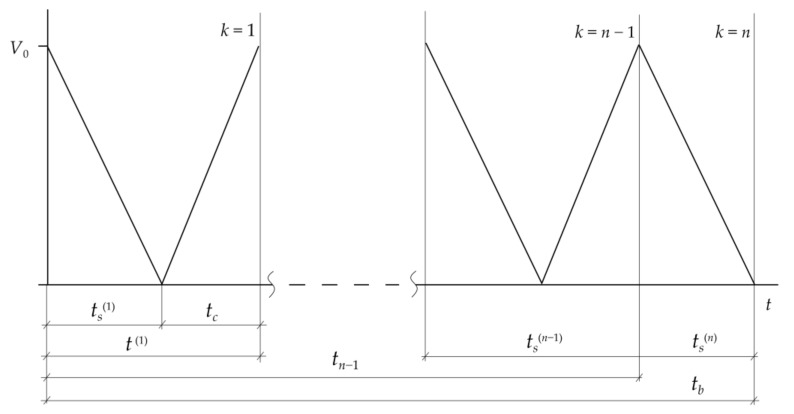
Scheme of sliding velocity variations during repetitive short-term (RST) braking mode.

A braking system operating in a repetitive short-term (RST) mode is considered. This intermittent braking mode comprised the sequential performance of n−1 full cycles and the final (*n*th) discontinuous cycle (Figure 1). Each of the full cycles consisted of two stages. The first one was braking, where the linear velocity of sliding, $V^{\left(k\right)}$, decreased from the initial value, $V_{0}$, to zero at time moments $t=t_{s}^{\left(k\right)},\;k=1,2,\ldots ,\;n-1$. Immediately after the standstill, the second phase of the cycle began, which was acceleration with the released brake. During this stage the velocity increased to initial value $V_{0}$ at time $t=t_{c}$.

Thus, the duration of one full cycle was $t_{k}=t_{s}^{\left(k\right)}+t_{c}$, k=1,2,…, n−1, and the overall time of all full braking cycles was $t_{n-1}=\sum_{k=1}^{n-1}t_{k}$. The last interrupted cycle was performed with the applied brake, so speed $V^{\left(n\right)}$ dropped from initial value $V_{0}$ to zero at stop moment $t=t_{s}^{\left(n\right)}$. Thus, the total time of the RST operation mode of braking was $t_{b}=t_{n-1}+t_{s}^{\left(n\right)}$.

Braking phases are accompanied by intense frictional heating of the friction pair elements (disc-pad, drum-shoe, etc.). Because of high temperature gradients, the friction coefficient and thermo-mechanical properties of friction pair elements can differ significantly at the initial phase and after braking. Hence, the assumption is made that, during the *k*th braking, friction coefficient f, thermal conductivities $K_{l}$, specific heat capacities $c_{l}$, densities $\rho_{l}$ and Brinell hardness $HB_{l}$ of friction pair materials l=1, 2 depend on temperature T in the form of, Equations (1) and (2):

(1) $$f(T)=f_{0}f^{*}(T),$$

(2) $$K_{l}(T)=K_{l,0}K_{l}^{*}(T),c_{l}(T)=c_{l,0}c_{l}^{*}(T),\;\rho_{l}(T)=\rho_{l,0}\rho_{l}^{*}(T),\;HB_{l}(T)=HB_{l,0}B_{l}^{*}(T),$$

Where Equations (3)–(8),

(3) $$f_{0}=f(T_{0}),\;K_{l,0}=K_{l}(T_{0}),\;c_{l,0}=c_{l}(T_{0}),\;\rho_{l,0}=\rho_{l}(T_{0}),\;HB_{l,0}=HB_{l}(T_{0}),$$

(4) $$f^{*}(T)=f_{1}+\frac{f_{2}}{{\left[f_{3}(T-f_{4})\right]}^{2}+1}+\frac{f_{5}}{{\left[f_{6}(T-f_{7})\right]}^{2}+1},$$

(5) $$K_{l}^{*}(T)=K_{l,1}+\frac{K_{l,2}}{{\left[K_{l,3}(T-K_{l,4})\right]}^{2}+1}+\frac{K_{l,5}}{{\left[K_{l,6}(T-K_{l,7})\right]}^{2}+1},$$

(6) $$c_{l}^{*}(T)=c_{l,1}+\frac{c_{l,2}}{{\left[c_{l,3}(T-c_{l,4})\right]}^{2}+1}+\frac{c_{l,5}}{{\left[c_{l,6}(T-c_{l,7})\right]}^{2}+1},$$

(7) $$\rho_{l}^{*}(T)=\rho_{l,1}+\frac{\rho_{l,2}}{{\left[\rho_{l,3}(T-\rho_{l,4})\right]}^{2}+1}+\frac{\rho_{l,5}}{{\left[\rho_{l,6}(T-\rho_{l,7})\right]}^{2}+1},$$

(8) $$HB^{*}(T)=HB_{1}+\frac{HB_{2}}{{\left[HB_{3}(T-HB_{4})\right]}^{2}+1}+\frac{HB_{5}}{{\left[HB_{6}(T-HB_{7})\right]}^{2}+1},$$

where $T_{0}$—initial temperature, $f_{j}$, $K_{l,j}$, $c_{l,j}$, $HB_{l,j}$, $\rho_{l,j}$, l=1,2; j=1,2,…,7 are parameters of the experimental data approximations [26]. Here and below, subscripts l=1 and l=2 refer to quantities associated with the primary element (disc, drum, etc.) and friction lining (pad, shoe, etc.), respectively.

The remaining assumptions of the calculation model are as follows:

1. At the initial moment of each braking phase, the friction element is pressed against the primary element contact surface with uniform pressure p, which exponentially increases with time t, from zero to nominal value $p_{0}$, Equation (9):
  (9) $$p(t)=p_{0}p^{*}(t),\;p^{*}(t)=1-e^{-\frac{t}{t_{i}}},\;0\leq t\leq t_{s}^{\left(k\right)},k=1,2,\ldots ,n,$$
  where $t_{i}$—time of pressure increase.
2. At the initial moment of the kth cycle of braking, the distribution of temperature in the tribosystem is homogeneous and equal to the averaged volumetric temperature of friction pair $T_{0}^{\left(k\right)}$;
3. As a result of the friction forces acting on the contact area of friction pair elements, heat is generated and absorbed by these elements in the normal directions of their friction surfaces;
4. The thermal contact of friction pair elements is perfect. In other words, the sum of heat flux intensities directed into friction elements, is equal to the specific friction power, and the temperatures of its contact areas are equal.
5. During the subsequent braking phases, the free surfaces of the brake system are adiabatic and during the acceleration stages, unforced convection cooling takes place.

The applied contact pressure (10) causes a reduction of sliding velocity $V^{\left(k\right)}$ during the *k*th braking, according to the following relation [25], Equation (10):

(10) $$V^{\left(k\right)}(t)=V_{0}\;V^{*(k)}(t),\;V^{*(k)}(t)=1-\frac{t}{t_{s,0}^{\left(k\right)}}+p^{*}(t)\frac{t_{i}}{t_{s,0}^{\left(k\right)}},\;0\leq t\leq t_{s}^{\left(k\right)},\;k=1,2,\ldots ,n,$$

Where, Equation (11),

(11) $$t_{s,0}^{\left(k\right)}=\frac{2W_{0}}{q_{0}^{\left(k\right)}A_{a}},$$

$W_{0}$—initial kinetic energy of the system, which depends on the mass of the vehicle and its velocity at the moment just before the brake applications, $q_{0}^{\left(k\right)}$—nominal specific friction power, $A_{a}$—nominal contact surface area, usually determined by the dimensions of the contact surface of friction element. Braking time $t_{s}^{\left(k\right)}$ is obtained from the stop condition, Equation (12):

(12) $$V^{*(k)}(t_{s}^{\left(k\right)})=0.$$

In case of the immediate ($t_{i}\to 0$) achievement of nominal pressure value $p_{0}$ in the Equation (9) from Equation (10) we obtain the linear drop in speed, Equation (13):

(13) $$V^{*(k)}(t)=1-\frac{t}{t_{s,0}^{\left(k\right)}},\;0\leq t\leq t_{s,0}^{\left(k\right)}.$$

Thus, parameter $t_{s,0}^{\left(k\right)}$ (11) is the time of braking with constant deceleration.

In the phases of acceleration, the velocity increases linearly with time, Equation (14):

(14) $$V^{*(k)}(t)=\frac{t-t_{s}^{\left(k\right)}}{t_{c}},\;t_{s}^{\left(k\right)}\leq t\leq t_{k},\;k=1,2,\ldots ,n.$$

The evolution of maximum temperature $T_{\max}^{\left(k\right)}$ on the friction surface during *k*th braking was sought in the form of [9,15]:

(15) $$T_{\max}^{\left(k\right)}(t)=T_{m}^{\left(k\right)}(t)+T_{f}^{\left(k\right)}(t),\;0\leq t\leq t_{s}^{\left(k\right)},\;k=1,2,\ldots ,n,.$$

where $T_{m}^{\left(k\right)}$—mean temperature of the nominal contact surface and $T_{f}^{\left(k\right)}$—average temperature of the real contact area (flash temperature).

## 3. Solution to the Problem

### 3.1. Heat Generation on the Nominal Contact Surface

Taking into account the above assumptions, to find component $T_{m}^{\left(k\right)}$ in the relation (16), we used the a calculation scheme of frictional contact of two different semi-infinite bodies, 0≤z<∞ (l=1) and −∞<z≤0 (l=2), sliding against each other with velocity $V^{\left(k\right)}(t)$ (10)–(13). Initiated by frictional heating during kth braking, we found transient temperature field $T^{\left(k\right)}(z,t)$ in this system from the solution to the following boundary-value problem of heat conduction, Equations (16)–(20):

(16) $$\frac{\partial^{2}T^{\left(k\right)}(z,t)}{\partial z^{2}}=\frac{1}{k_{1,0}^{\left(k\right)}}\frac{\partial T^{\left(k\right)}(z,t)}{\partial t},\;0<z<\infty ,\;0<t\leq t_{s}^{\left(k\right)},$$

(17) $${\left.K_{2,0}^{\left(k\right)}\frac{\partial T^{\left(k\right)}(z,t)}{\partial z}\right|}_{z=0^{-}}{\left.-K_{1,0}^{\left(k\right)}\frac{\partial T^{\left(k\right)}(z,t)}{\partial z}\right|}_{z=0^{+}}=q^{\left(k\right)}(t),\;0<t\leq t_{s}^{\left(k\right)},$$

(18) $$T^{\left(k\right)}\left(0^{+},t\right)=T^{\left(k\right)}\left(0^{-},t\right)\equiv T_{m}^{\left(k\right)}\left(t\right),\;0<t\leq t_{s}^{\left(k\right)},$$

(19) $$T^{\left(k\right)}\left(z,t\right)\to T_{0}^{\left(k\right)},\;\left|z\right|\to \infty ,\;0<t\leq t_{s}^{\left(k\right)}$$

(20) $$T^{\left(k\right)}(z,0)=T_{0}^{\left(k\right)},\;\;\;\left|z\right|<\infty ,\;k=1,2,\ldots ,n$$

Where, Equations (21)–(23),

(21) $$q^{\left(k\right)}(t)=q_{0}^{\left(k\right)}q^{*(k)}(t),\;q_{0}^{\left(k\right)}=f_{0}^{\left(k\right)}p_{0}V_{0},\;q^{*(k)}(t)=p^{*}(t)V^{*(k)}(t)$$

(22) $$k_{l,0}^{\left(k\right)}=\frac{K_{l,0}^{\left(k\right)}}{\rho_{l,0}^{\left(k\right)}c_{l,0}^{\left(k\right)}},\;l=1,2,$$

(23) $$f_{0}^{\left(k\right)}=f(T_{0}^{\left(k\right)}),\;K_{l,0}^{\left(k\right)}=K_{l}(T_{0}^{\left(k\right)}),\;c_{l,0}^{\left(k\right)}=c_{l}(T_{0}^{\left(k\right)}),\;\rho_{l,0}^{\left(k\right)}=\rho_{l}(T_{0}^{\left(k\right)}).$$

Averaged volumetric temperature $T_{0}^{\left(k\right)}$ of a braking system before *k*th braking was calculated from the formulas, Equations (24)–(26) [9,16]:

(24) $$T_{0}^{\left(k\right)}=\frac{T_{0,0}^{\left(k\right)}+T_{0,1}^{\left(k\right)}}{2},\;k=1,2,\ldots ,n,.$$

(25) $$T_{0,i}^{\left(k\right)}=T_{0}+\frac{\gamma_{i}W_{0}}{2G\;c_{1,i}}\left(\frac{e^{-\alpha_{i}t_{c}}-e^{-k\alpha_{i}t_{c}}}{1-e^{-\alpha_{i}t_{c}}}\right),\;i=0,\;1,$$

(26) $$\gamma_{i}=\frac{\sqrt{K_{1,i}\rho_{1,i}c_{1,i}}}{\sqrt{K_{1,i}\rho_{1,i}c_{1,i}}+\sqrt{K_{2,i}\rho_{2,i}c_{2,i}}}$$

Where, Equations (27)–(29),

(27) $$\gamma_{i}=\frac{\sqrt{K_{1,i}\rho_{1,i}c_{1,i}}}{\sqrt{K_{1,i}\rho_{1,i}c_{1,i}}+\sqrt{K_{2,i}\rho_{2,i}c_{2,i}}}$$

(28) $$\alpha_{i}=\frac{hA_{vent}}{G\;c_{1,i}}$$

(29) $$K_{l,1}=K_{l}(T_{0,0}^{\left(k\right)}),\;c_{l,1}=c_{l}(T_{0,0}^{\left(k\right)}),\;\rho_{l,1}=\rho_{l}(T_{0,0}^{\left(k\right)}),$$

h—heat transfer coefficient, G, $A_{vent}$—weight and total surface area of the primary element of friction pair, respectively; values $K_{l,0}$, $c_{l,0}$, $\rho_{l,0}$, l=1, 2 are defined by Equation (3). For the first braking cycle (k=1)), from relations (25) and (26), it follows that $T_{0}^{\left(1\right)}=T_{0}$.

Exact solution to the boundary-value problem of heat conduction, (17)–(22), during single braking was obtained in [27]. Generalizing this solution to the considered case of repetitive braking, the sought temperature on the friction surface was written in the form, Equation (30):

(30) $$T_{m}^{\left(k\right)}(t)=T_{0}^{\left(k\right)}+T_{m,0}^{\left(k\right)}T_{m}^{*(k)}(t)\;,0\leq t\leq t_{s}^{\left(k\right)}\;,k=1,2,\ldots ,n$$

where, Equations (31)–(37),

(31) $$T_{m}^{*(k)}(t)=\gamma_{0}^{\left(k\right)}\sqrt{\frac{t}{t_{s,0}^{\left(k\right)}}}\left[\left(1+\frac{t_{i}}{t_{s,0}^{\left(k\right)}}-\frac{2t}{3t_{s,0}^{\left(k\right)}}\right)\frac{2}{\sqrt{\pi }}-\left(1+\frac{3t_{i}}{2t_{s,0}^{\left(k\right)}}-\frac{t}{t_{s,0}^{\left(k\right)}}\right)\;F\left(\sqrt{\frac{t}{t_{s,0}^{\left(k\right)}}}\right)+\frac{t_{i}}{t_{s,0}^{\left(k\right)}}\;F\left(\sqrt{\frac{2t}{t_{s,0}^{\left(k\right)}}}\right)\right]$$

(32) $$F(x)=\frac{2}{\sqrt{\pi }}\sum_{n=0}^{\infty }{\left(-1\right)}^{n}\frac{{\left(2x^{2}\right)}^{n}}{(2n+1)!!},\;0\leq x\leq 3,\;F(x)=\frac{2}{\sqrt{\pi }}\sum_{n=0}^{\infty }\frac{(2n-1)!!}{{\left(2x^{2}\right)}^{n+1}},\;x>3,$$

(33) $$\gamma_{0}^{\left(k\right)}=\frac{\sqrt{K_{1,0}^{\left(k\right)}\rho_{1,0}^{\left(k\right)}c_{1,0}^{\left(k\right)}}}{\sqrt{K_{1,0}^{\left(k\right)}\rho_{1,0}^{\left(k\right)}c_{1,0}^{\left(k\right)}}+\sqrt{K_{2,0}^{\left(k\right)}\rho_{2,0}^{\left(k\right)}c_{2,0}^{\left(k\right)}}},$$

(34) $$T_{m,0}^{\left(k\right)}=\frac{q_{0}^{\left(k\right)}a^{\left(k\right)}}{K_{1,0}^{\left(k\right)}}$$

(35) $$a^{\left(k\right)}=\max\{a_{l}^{\left(k\right)}\}$$

(36) $$a_{l}^{\left(k\right)}=\left\{\begin{array}{l} d_{l},\;\;a_{l,eff}^{\left(k\right)}\geq d_{l}\;,\\ a_{l,eff}^{\left(k\right)},\;\;a_{l,eff}^{\left(k\right)}<d_{l}\;, \end{array}\right.$$

(37) $$a_{l,eff}^{\left(k\right)}=\sqrt{3k_{l,0}^{\left(k\right)}t_{s,0}^{\left(k\right)}},$$

$d_{l}$, l=1, 2 thicknesses of the friction pair components.

The algorithm to find the mean temperature for the selected friction pair consisted in the subsequent performance of the following steps:

1. Based on the experimental data, by means of the approximation formulas (1)–(8), describe the thermal stability of friction f(T) and temperature dependencies of thermal $K_{l}(T)$, $c_{l}(T)$ and mechanical $\rho_{l}(T)$, $HB_{l}(T)$ properties of friction materials l=1,2;
2. Set the operation input parameters: $p_{0}$, $V_{0}$, $T_{0}$, $W_{0}$, n, $A_{a}$, h, G, $t_{i}$, $t_{c}$, $d_{l}$, $K_{l,0}$, $c_{l,0}$, $HB_{l,0}$, $\rho_{l,0}$, l=1,2;
3. Begin the first (k=1) braking cycle;
4. Establish the averaged volumetric temperature $T_{0}^{\left(k\right)}$ of the friction pair from Formulas (25)–(29);
5. Taking into account dependencies (1)–(8) calculate the values of friction coefficient $f_{0}^{\left(k\right)}$ and materials properties $K_{l,0}^{\left(k\right)}$, $\rho_{l,0}^{\left(k\right)}$, l=1,2 (25) in temperature $T_{0}^{\left(k\right)}$;
6. Determine braking time $t_{s}^{\left(k\right)}$ and temporal profile of velocity $V^{\left(k\right)}(t)$, $0\leq t\leq t_{s}^{\left(k\right)}$ from Equations (10)–(12) and (22);
7. Calculate the evolution of mean temperature on nominal contact surface $T_{m}^{*(k)}(t)$, $0\leq t\leq t_{s}^{\left(k\right)}$ (33)–(38);
8. Start the subsequent (k+1) cycle of braking and repeat the calculations, beginning from point 4). The calculation process ends when condition k=n is met.

### 3.2. Temperature of the Real Contact Region

Real friction surfaces of braking system elements are not perfectly smooth, they are characterized by significant roughness and waviness. Therefore, these elements are not in contact on an entire nominal friction area, but only in some parts of one, and consists of roughness waves. These waves form the so-called contour surface of contact, the area of which ($A_{c}^{\left(k\right)}$) changes during *k*th braking, according to the law, Equations (38) and (39) [28]:

(38) $$A_{c}^{\left(k\right)}(t)=A_{a}{\left[\frac{p(t)b_{0}^{\nu -1}}{HB_{\min}^{\left(k\right)}(t)}\right]}^{\frac{1}{\nu +1}},\;0\leq t\leq t_{s}^{\left(k\right)},$$

(39) $$HB_{\min}^{\left(k\right)}(t)=\min\{HB_{1}[T_{m}^{\left(k\right)}(t)],HB_{2}[T_{m}^{\left(k\right)}(t)]\}$$

where p, $T_{m}^{\left(k\right)}$—pressure (9) and mean temperature (30)–(37) on the nominal contact surface with area $A_{a}$, $b_{0}^{}$, ν—parameters of the reference curve for the harder element of the friction pair materials. Usually this is the material of a primary element (disc, drum etc.). Pressure $p_{c}^{\left(k\right)}$, on contour area $A_{c}^{\left(k\right)}$ (38), (39) was found from the relation, Equation (40):

(40) $$p_{c}^{\left(k\right)}(t)=p(t)\frac{A_{a}}{A_{c}^{\left(k\right)}(t)},\;0\leq t\leq t_{s}^{\left(k\right)}$$

In a typical braking system, the friction linings are made of more deformable and less durable material than the primary element material. Thus, we assume that:

1. Asperities have the spherical shape and are located on the surface of the harder and stiffer primary element, while the friction lining surface is smooth.
2. Plastic roughness deformation mechanism takes place. This means that the contact of a single asperity with the friction lining surface lasts until its material becomes plastic due to a rapid increase in temperature and the appearance of significant thermal stresses.
3. Before coming into contact with the friction lining, the temperature of asperity does not change along its height and is equal to the mean temperature of nominal contact area $T_{m}^{\left(k\right)}$.

Contact of a single asperity with a smooth friction lining surface creates the real region of contact with a diameter as follows Equation (41) [29]:

(41) $$d_{r}^{\left(k\right)}(t)={\left(\frac{8r_{a}{}_{v}h_{\max}}{\nu }\right)}^{\frac{1}{2}}{\left[\frac{p_{c}^{\left(k\right)}(t)}{HB_{\min}^{\left(k\right)}(t)b_{0}}\right]}^{\frac{1}{2\nu }},\;0\leq t\leq t_{s}^{\left(k\right)},$$

where $r_{av}$—averaged radius of the asperities rounding and $h_{\max}$—maximum height of the roughness. Total area $A_{r}^{\left(k\right)}$ of the real contact region is related to area $A_{a}$ of the nominal contact surface according to the formula, Equation (42):

(42) $$A_{r}^{\left(k\right)}(t)=A_{a}\frac{p(t)}{HB_{\min}^{\left(k\right)}(t)},\;0\leq t\leq t_{s}^{\left(k\right)}.$$

Evolution of temperature $T_{f}^{\left(k\right)}$ on the real contact region with area $A_{r}^{\left(k\right)}$ (42) (flash temperature) was determined from the following Equation (43) [26,30]:

(43) $$T_{f}^{\left(k\right)}(t)=\left(1+\frac{1}{\sqrt{2}}\right)\frac{f_{m}^{\left(k\right)}(t)p(t)V^{\left(k\right)}(t)A_{a}d_{r}^{\left(k\right)}(t)}{\;A_{r}^{\left(k\right)}(t)[4K_{1,m}^{\left(k\right)}(t)+\sqrt{\pi V^{\left(k\right)}(t)\;d_{r}^{\left(k\right)}(t)\;K_{2,m}^{\left(k\right)}(t)\;c_{2,m}^{\left(k\right)}(t)\;\rho_{2,m}^{\left(k\right)}(t)}]},\;0\leq t\leq t_{s}^{\left(k\right)},$$

Where Equation (44):

(44) $$f_{m}^{\left(k\right)}\left(t\right)\equiv f\left[T_{m}^{\left(k\right)}\left(t\right)\right],\;K_{l,m}^{\left(k\right)}\left(t\right)\equiv K_{l}\left[T_{m}^{\left(k\right)}\left(t\right)\right],\;c_{l,m}^{\left(k\right)}\left(t\right)\equiv c_{l}\left[T_{m}^{\left(k\right)}\left(t\right)\right],\;\rho_{l,m}^{\left(k\right)}\left(t\right)\equiv \rho_{l}\left[T_{m}^{\left(k\right)}\left(t\right)\right],\;l=1,2.$$

Summarizing, another scheme was proposed for determining the flash temperature evolution during *k*th braking:

1. On the basis of the friction surface profiles of the primary friction element, the average values of parameters $b_{0}^{}$, ν, $r_{av}$, $h_{\max}$ characterizing the roughness shape and their distribution along the height were calculated in the longitudinal and transverse directions.
2. Knowing the temporal profile of mean temperature on nominal contact surface $T_{m}^{\left(k\right)}$ (30)–(37), by means of approximation functions (1)–(8), the evolutions of $HB_{l,m}^{\left(k\right)}$ (39) and $f_{m}^{\left(k\right)}$, $K_{l,m}^{\left(k\right)}$, $c_{l,m}^{\left(k\right)}$, $\rho_{l,m}^{\left(k\right)}$, l=1,2 (44) were established.
3. Variations of contour contact area $A_{c}^{\left(k\right)}$ (38), (39) and contour pressure $p_{c}^{\left(k\right)}$ (40) during braking were determined, taking into account pressure profile p (9).
4. Changes of diameter $d_{r}^{\left(k\right)}$ (41) and total area of real contact $A_{r}^{\left(k\right)}$ (42) in time were established.
5. Evolution of flash temperature $T_{f}^{\left(k\right)}$ (43) was calculated, taking into account velocity temporal profile $V^{\left(k\right)}$ (10)–(12).

## 4. Numerical Analysis

**Figure 2:**
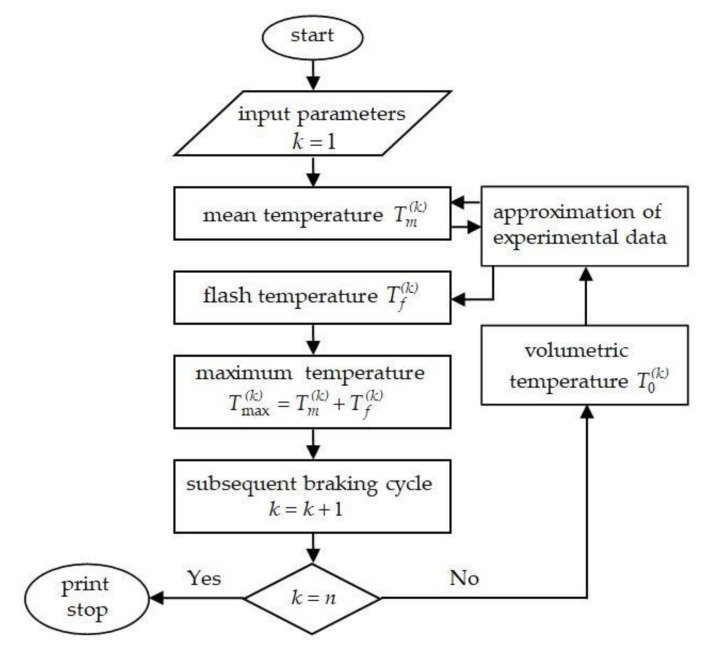
Flowchart of the calculation procedure.

Based on the analytical model proposed above, the temperature generated during RST braking mode was studied for two tribosystems: disc and drum brakes. The temperature field of the disc brake system was analyzed using the finite differences method in [16]. Additionally, the corresponding evolutions of temperature in the drum brake were presented in [9], where the calculations were performed on the basis of approximate solutions to the thermal problem of friction for two strips and experimental data. Results of calculations presented below were obtained for the same friction materials and operational parameters as the above-mentioned studies. In both systems, the RST braking mode was considered, which includes 4 cycles (n=4): three full cycles (braking-acceleration) and the last interrupted cycle (braking). The brake disc is made of cast iron ChNMKh, and brake pads—were manufactured from cermet FMC-11. In the second system, the drum brake was made of 30KhHSA steel and the brake shoes were manufactured with retinax FC-16L. The sintered cermet friction material FMC-11 contains 64% Fe, 15% Cu, 3% SiO_2_, 6% BaSO_4_, 3% asbestos and 9% graphite. Retinax FC-16L is a composite based on phenol-formaldehyde resins and reinforced with brass shavings [21]. The thermophysical and mechanical properties of materials and friction coefficients of selected pairs at the initial temperature are included in Table 1. The methodology of calculations adopted in the present study is shown in Figure 2.

**Figure 3:**
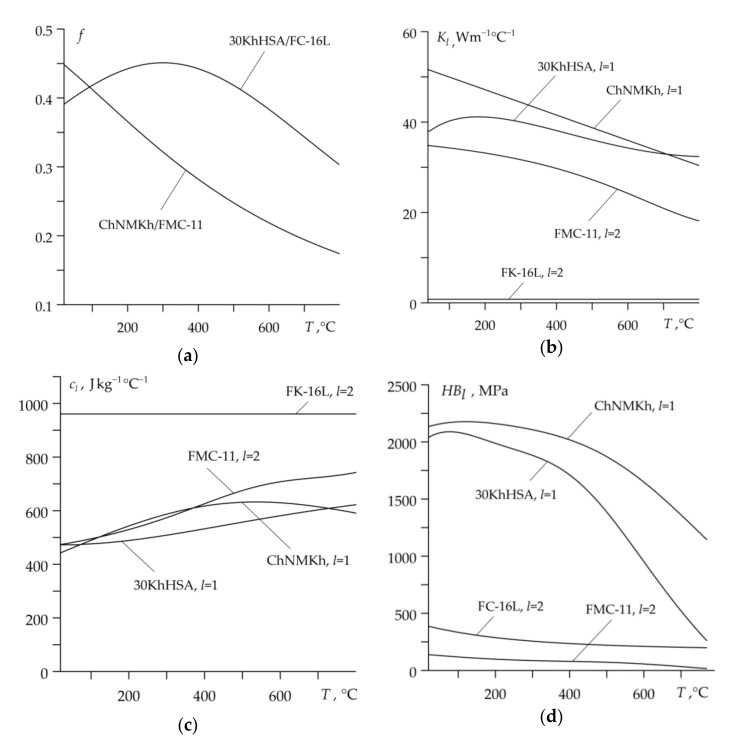
Graphs of functions approximating the experimental data of the dependence on temperature: (**a**) coefficient of friction f; (**b**) thermal conductivity $K_{l}$; (**c**) specific heat capacity $c_{l}$; (**d**) Brinell hardness $HB_{l}$, l=1,2.

Experimental data of frictional thermal stability and property variations under different temperature conditions of friction materials ChNMKh/FMC-11 and 30KhHSA/FC-16L are included in [9,26]. The values of coefficients in the functions (4)–(8), which approximate these data, are presented in Table 2. In this paper, it was assumed that density $\rho_{l}$ of all selected materials changes slightly under temperature variations, so Equation (7) takes the form $\rho_{l}^{*}(T)=1$, l=1, 2. Graphs of functions f(T) (4), $K_{l}(T)$ (5), $c_{l}(T)$ (6) and $HB_{l}(T)$, l=1, 2 (8) are presented in Figure 3.

### 4.1. Disc Brake System

The input values of operating parameters [15,16]: $p_{0}=1.47\;\mathrm{MPa}$, $V_{0}=27.78\;m\;s^{-1}$, $W_{0}=392.1\;\mathrm{kJ}$, $A_{a}=4.047\cdot 10^{-2}\;m^{2}$, $A_{vent}=4.44\cdot 10^{-2}\;m^{2}$, G=1.58 kg, $h=100\;\;{\mathrm{Wm}}^{-2}K^{-1}$, $d_{1}=5.5\;\mathrm{mm}$, $d_{2}=10\;\mathrm{mm}$, $t_{i}=0.5\;s$, $t_{c}=5\;s$, $r_{av}=450\;\mu m$, $h_{\max}=2.5\;\mu m$, $b_{0}=1.0$, ν=2.1. Due to the geometric symmetry of the disc brake system with respect to the center plane of the disc, to determine the temperature field we consider the tribosystem, which consists of disc replacement and the pad with thicknesses of $d_{l}$, l=1,2, respectively. The thickness of the disc replacement element $d_{1}$ is equal to the half of the real disc dimension.

**Figure 4:**
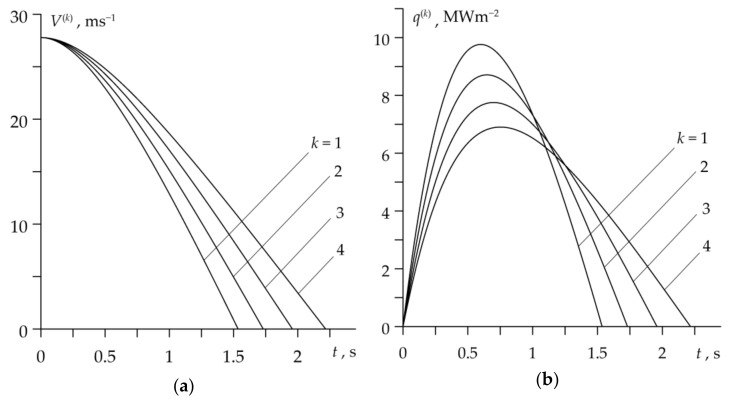
Changes in: (**a**) velocity $V^{\left(k\right)}$ and (**b**) specific friction power $q^{\left(k\right)}$ during k=1,2,3,4 braking applications of a disc brake system.

The temporal profiles of velocity $V^{\left(k\right)}$ (10) and specific friction power $q^{\left(k\right)}$ (22) during four considered braking cycles are presented in Figure 4. Except for the short initial period of braking, when the pressure grows, the sliding speed linearly decreases with time and the stop time is longer with every subsequent cycle (Figure 4a). The specific friction power increases at the beginning of each braking cycle until reaching the maximum value, and then decreases to zero at the stop moment (Figure 4b), which is characteristic for the so-called rational braking mode [18,19]. It should be noted that the friction work performed at each braking cycle (the area under each of the four curves in Figure 4b) was the same and equal to the initial kinetic energy of the system $W_{0}=392.1\;\mathrm{kJ}$.

**Figure 5:**
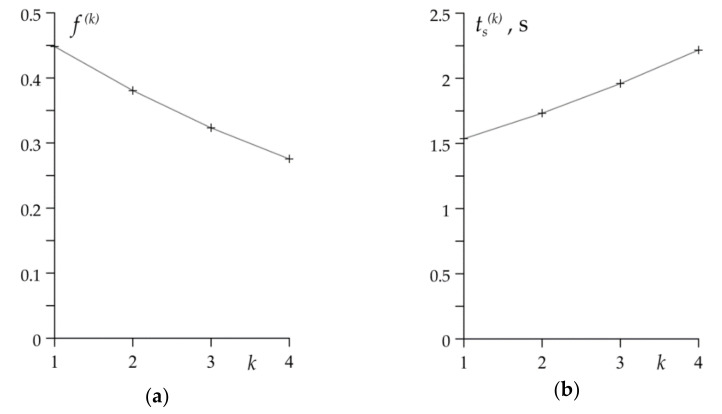
The values of: (**a**) friction coefficient $f_{0}^{\left(k\right)}$; (**b**) stop time $t_{s}^{\left(k\right)}$ for disc brake in each braking cycle k=1,2,3,4.

The coefficient of friction $f_{0}^{\left(k\right)}$ (24) decreases with every subsequent braking from 0.45 during the first cycle to 0.28 during the fourth, last braking (Figure 5a). The drop of the friction coefficient value causes the elongation of braking stage $t_{s}^{\left(k\right)}$—its values are equal to 1.54 s, 1.73 s, 1.96 s and 2.22 s, respectively for k=1,2,3,4 (Figure 5b).

**Figure 6:**
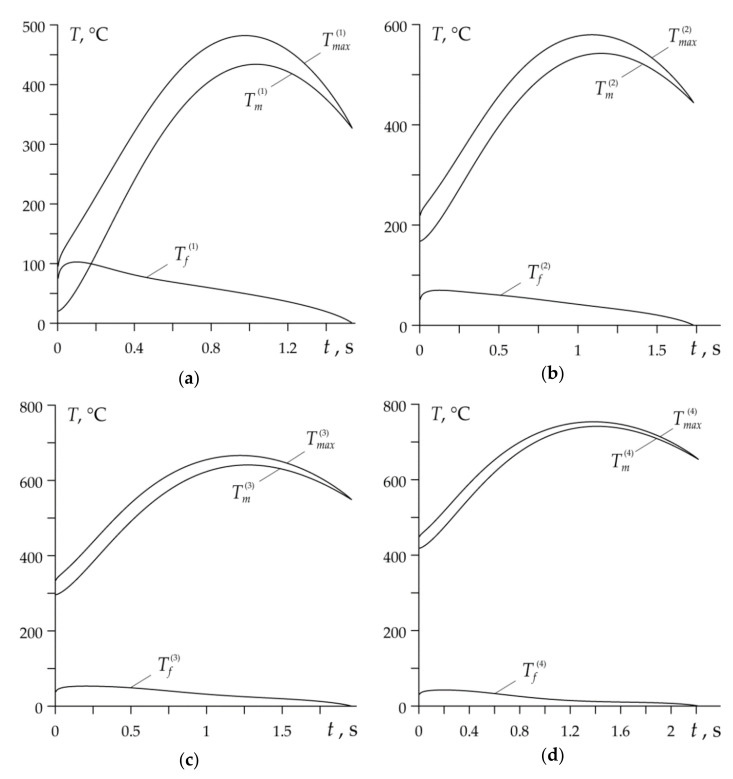
Evolutions of the mean $T_{m}^{\left(k\right)}$, flash $T_{f}^{\left(k\right)}$ and maximum $T_{\max}^{\left(k\right)}$ temperatures for disc brake during each cycle: (**a**) *k* = 1; (**b**) *k* = 2; (**c**) *k* = 3; (**d**) *k* = 4.

Variations of mean temperature $T_{m}^{\left(k\right)}$ (30), flash temperature $T_{f}^{\left(k\right)}$ (43) and maximum temperature $T_{\max}^{\left(k\right)}$ (15) during each braking cycle of the process are shown in Figure 6.

Evolutions of mean temperature on the nominal contact area during every braking stage correspond to the temporal profiles of specific friction power, presented in Figure 4b. At the beginning of each braking cycle, the mean temperature $T_{m}^{\left(k\right)}$ growth takes place until maximum value is reached, and then the temperature drop to the standstill occurs. According to the adapted plastic roughness deformation mechanism, the flash temperature reaches significant values at the initial moments of the braking stages, when the contact area of the pad with the disc is relatively “cold”. Subsequent heating of this area causes a rapid decrease in the flash temperature $T_{f}^{\left(k\right)}$. The mean temperature of the friction surface has a decisive influence on the time profile and the maximum temperature value. With each subsequent braking, the highest values of $T_{m}^{\left(k\right)}$ and $T_{\max}^{\left(k\right)}$ increase, while the $T_{f}^{\left(k\right)}$ decreases.

**Figure 7:**
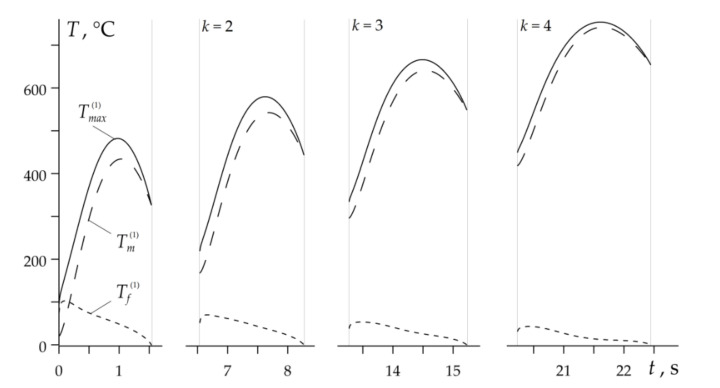
Variations of the maximum $T_{\max}^{\left(k\right)}$ (solid lines), the mean $T_{m}^{\left(k\right)}$ (dashed lines) and the flash $T_{f}^{\left(k\right)}$ (dotted lines) temperatures during disc brake RST mode.

Temporal profiles of $T_{m}^{\left(k\right)}$, $T_{f}^{\left(k\right)}$ and $T_{\max}^{\left(k\right)}$ during the entire RST braking mode are presented in Figure 7. The combined time of all four braking cycles duration is 7.45 s and with account of the three stages of acceleration 15 s, gives the total time of the whole RST braking mode in the disc brake system $t_{b}=22.45\;s$.

### 4.2. Drum Brake System

The values of input parameters of braking in the RST mode by friction pair consisting of the drum (l=1) and brake shoe (l=2), were adapted [9]: $p_{0}=0.44\;\mathrm{MPa}$, $V_{0}=11.5\;m\;s^{-1},$ $W_{0}=215.7\;\mathrm{kJ}$, $A_{a}=A_{c}=3.85\cdot 10^{-2}\;m^{2}$, $A_{vent}=15\cdot 10^{-2}\;m^{2}$, G=5.5 kg, $h=80\;\;{\mathrm{Wm}}^{-2}K^{-1}$, $d_{1}=10\;\mathrm{mm}$, $d_{2}=18\;\mathrm{mm}$, $t_{i}=0.5\;s$, $t_{c}=25\;s$, $r_{av}=500\;\mu m$, $h_{\max}=4.5\;\mu m$, $b_{0}=1.0$, ν=2.2.

**Figure 8:**
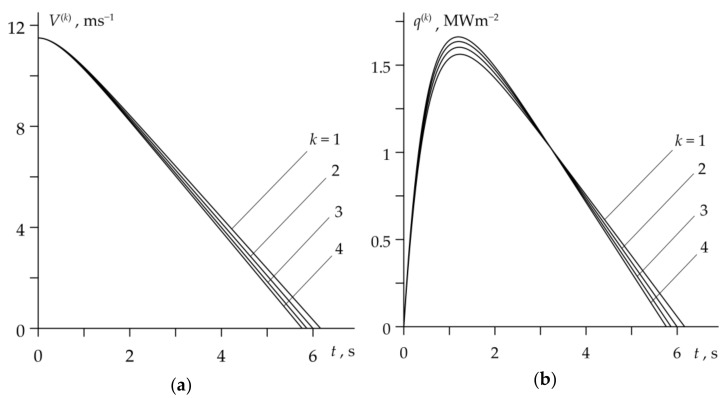
Changes in time during RST mode of the drum brake: (**a**) velocity $V^{\left(k\right)}$; (**b**) specific friction power $q^{\left(k\right)}$, k=1,2,3,4.

The evolutions of sliding velocity and specific friction power for the drum brake are presented in Figure 8. There are some similarities with the corresponding profiles for a disc brake, which are shown in Figure 4. Sliding velocities also have short time of nonlinear drop (Figure 8a), and the profiles of specific friction power have a local maximum, shifted closer to the beginning of the braking period in this case (Figure 8b). Unlike the disc system, however, the duration of the braking stage in the drum brake is shortened and the maximum value of the specific friction power decreases with each subsequent braking.

**Figure 9:**
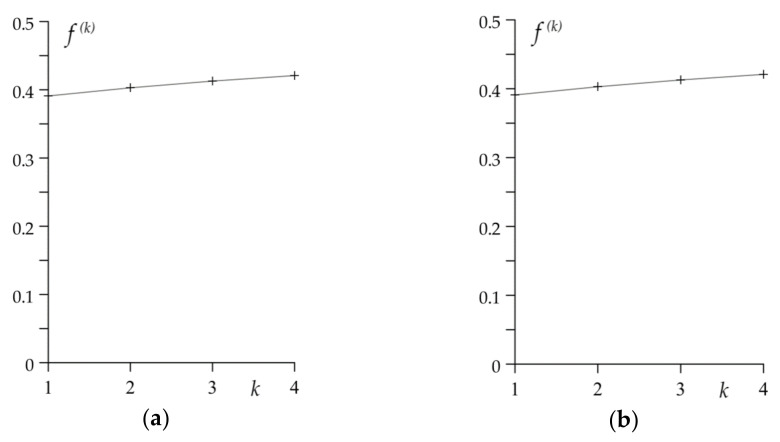
The values of: (**a**) coefficient of friction $f_{}^{\left(k\right)}$; (**b**) stopping time $t_{s}^{\left(k\right)}$ for the drum brake in each braking cycle k=1,2,3,4.

In Figure 8a the shortening of the time of subsequent braking can be noticed, which is related to the corresponding changes in the friction coefficient: in the considered drum system its value during the first braking is 0.39 and increases linearly to 0.42 during the fourth braking (Figure 9a). The respective stop times are equal to 6.17 s and 5.77 s (Figure 9b).

**Figure 10:**
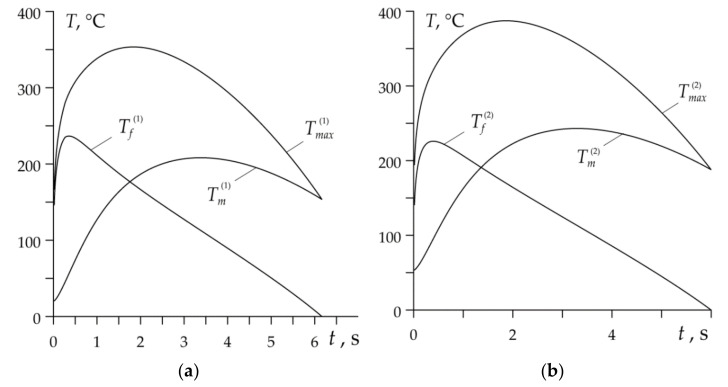

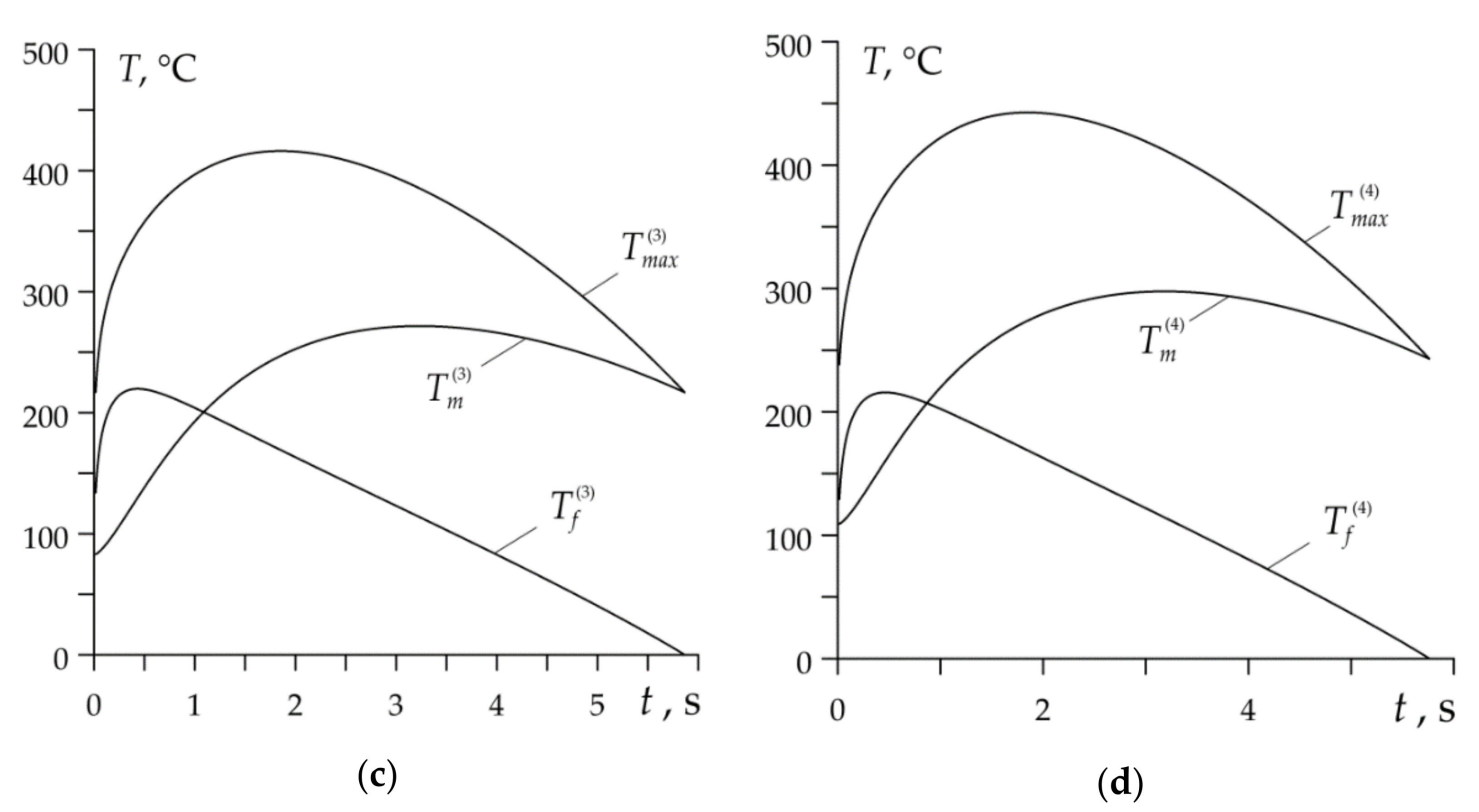
Evolutions of the mean $T_{m}^{\left(k\right)}$, flash $T_{f}^{\left(k\right)}$ and maximum $T_{\max}^{\left(k\right)}$ temperatures for drum brake during each cycle: (**a**) *k* = 1; (**b**) *k* = 2; (**c**) *k* = 3; (**d**) *k* = 4.

Much smaller changes in the specific friction power time profiles during each braking (Figure 8b) than in the case of a disc brake result in the fact that the temperature evolution in the drum brake during each braking stage also differs much less (Figure 10). On the other hand, the contribution of the mean temperature $T_{m}^{\left(k\right)}$ and the flash temperature $T_{f}^{\left(k\right)}$ to the value of the maximum temperature $T_{\max}^{\left(k\right)}$ in the drum brake are completely different than in the disc brake. In the latter, as noted above, the time profile of the maximum temperature was primarily shaped by the mean temperature on the friction surface. In the drum brake, both components $T_{m}^{\left(k\right)}$ and $T_{f}^{\left(k\right)}$ show a significant influence on evolution and values of $T_{\max}^{\left(k\right)}$. The decisive factor in the initial braking phase is the flash temperature, and in the final stage—the mean temperature of the drum-brake shoe contact area.

**Figure 11:**
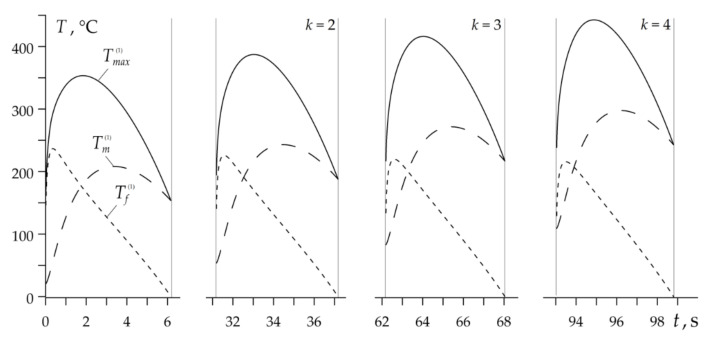
Evolutions of the maximum $T_{\max}^{\left(k\right)}$ (solid lines), mean $T_{m}^{\left(k\right)}$ (dashed lines) and flash $T_{f}^{\left(k\right)}$ (dotted lines) temperatures during drum brake RST mode.

The changes in the temperature with time during RST braking in the drum brake are presented in Figure 11. Duration of four braking cycles was equal to 23.81 s, three accelerations 75 s, so the total RST operation mode lasted 98.81 s. As in the case of the disc brake, with each subsequent braking, the highest values of the mean and the maximum temperatures increase, whereas the flash temperatures decrease. However, these relations are not as noticeable as in the disc brake. One of the reasons for such relatively small changes in temperature can result from much longer (five times) cooling phase during vehicle acceleration.

Values of the friction coefficient $f^{\left(k\right)}$, braking time $t_{s}^{\left(k\right)}$, volumetric temperature $T_{0}^{\left(k\right)}$ and the highest values of the mean temperature $T_{m}^{\left(k\right)}$, flash temperature $T_{f}^{\left(k\right)}$ and maximum temperature $T_{\max}^{\left(k\right)}$ during performing each of the four cycles are included in Table 3.

## 5. Summary of the Results and Discussion

The analytical model to determine the maximum temperature reached as a result of friction during the repetitive short-term operating mode of the braking systems, consisting of the n braking and acceleration cycles was proposed. The thermal sensitivity of the materials of the friction pairs and the temperature dependence of the friction coefficient were taken into account. According to the Chichinadze’s summation hypothesis, the maximum temperature $T_{\max}^{\left(k\right)}$ during each braking (k=1,2,…,n) was searched for as the sum of the mean temperature $T_{m}^{\left(k\right)}$ on the nominal contact area and the average temperature $T_{f}^{\left(k\right)}$ of the real contact area (flash temperature). The numerical analysis was carried out for two systems: disc brake (cast iron/cermet) and drum brake (steel/retinax) with n=4:

- Dependence of the friction coefficient on temperature (thermal stability curve) shows a significant influence on the time profiles of the velocity, specific friction power and maximum temperature. The coefficient of friction, which decreases with increasing temperature in the disc brake system, results in elongation of each subsequent braking stage and growth of the maximum values of the specific friction power. The effect of the friction coefficient increase, under temperature increase to about 300 °C in the drum brake system, is the reduction of the braking time and the increase of the maximum values of the specific friction power.
- In the disc brake system operating in heavy mode, the evolution of temperature and its maximum values $T_{\max}^{\left(k\right)}$ are determined by the mean temperature $T_{m}^{\left(k\right)}$ on the nominal contact area. The contribution of the flash temperature $T_{f}^{\left(k\right)}$ to the maximum temperature is negligible.
- In the drum brake operating under light conditions, at the beginning of each braking stage, maximum temperature is determined mainly by the flash temperature, while at the end of braking it depends mostly from the mean temperature $T_{m}^{\left(k\right)}$.
- The results obtained by means of the proposed analytical model show satisfactory compliance with the relevant data obtained with the use of numerical methods, published in the scientific literature. In particular, the highest values of the maximum temperature $T_{\max}^{\left(k\right)}$ at the subsequent stages of braking k=1,2,3,4, found as a result of our calculations, are 482 °C, 560 °C, 666 °C and 753 °C (Table 3), and the corresponding data obtained in the article [16] are equal to 491 °C, 615 °C, 720 °C, 847 °C, respectively. The greatest relative percentage difference of the results occurred in the fourth stage and is equal to ≈11%. In the drum brake the maximum temperatures $T_{\max}^{\left(k\right)}$ determined by means of the proposed model, are equal to 353 °C, 387 °C, 416 °C and 443 °C (Table 3), and corresponding results presented in monograph [9] are 295 °C, 330 °C, 400 °C and 440 °C. The highest relative difference in outcomes occurs in the stage one and is equal to ≈21%. It should be noted that the mean temperature $T_{m}^{\left(k\right)}$ of the selected friction pair drum-brake shoe with similar input parameters during single braking (k=1) was analyzed with the use of the finite difference method in the article [32]. The highest value of the mean temperature on the nominal contact area of the drum-brake shoe obtained was equal to 210 °C [32], which is in good agreement with the value 280 °C presented in Table 3.

## 6. Conclusions

The main advantage of the proposed approach is the demonstration of the possibility of adapting numerous exact solutions to the linear thermal problems of friction existing in the scientific literature, to determine the maximum temperature of the brake not only during a single, but also during a repetitive operation mode. This allows for express estimation with sufficient accuracy, not only of the maximum temperature, but also of important braking characteristics such as variations of the sliding velocity and specific friction power, stopping time and braking distance during each stage of RST braking mode. It should be emphasized that the proposed calculation model is coupled—it allows to take into account the mutual influence of all of the above-mentioned characteristics in the braking process, by the temperature-dependent coefficient of friction. From the point of view of application possibilities, it is important that this model can be used for calculations not only for materials with stable thermo-physical properties, but also for thermally sensitive materials.

## Figures and Tables

**Table 1:** Properties of friction pair materials at the initial temperature $T_{0}=$ 20 °C [26].

Material	f	$K_{l}\;,\;{\mathrm{Wm}}^{-1}\;K^{-1}$	$c_{l}\;,\;{\mathrm{Jkg}}^{-1}\;K^{-1}$	$\rho_{l}\;,\;\mathrm{kg}\;m^{-3}$	$HB_{l}\;,\;\mathrm{MPa}$
ChNMKh	0.45	52.17	444.6	7100	2100
FMC-11		35	479	4700	137
30KhHSA	0.39	38	490	7800	2050
FC-16L		0.79	961	2500	392

**Table 2:** Coefficients in approximation functions (4)–(6) and (8) for considered materials [31].

Coefficients	Material	*i* = 1	*i* = 2	*i* = 3, ${}^{\circ }C^{-1}$$,\times 10^{3}$	*i* = 4, ${}^{\circ }C$	*i* = 5	*i* = 6, ${}^{\circ }C^{-1}$$,\times 10^{3}$	*i* = 7, ${}^{\circ }C$
$f_{i}$	ChNMKh/FMC-11	0.01	1.07	1.5	–250	0	0	0
	30KhHSA/FC-16L	0	1.1	0.0014	300	0	0	0
$K_{l,i}$	ChNMKh	–2.37	4.22	0.196	–2543	0	0	0
	FMC-11	1.125	–0.64	2.3	900	0	0	0
	30KhHSA	2.455	–1.58	0.86	847	–1.05	6.3	–163
	FC-16L	1	0	0	0	0	0	0
$c_{l,i}$	ChNMKh	–0.85	6.6	0.57	4903	1.37	1.2	443
	FMC-11	0.78	0.74	3.5	1059	0.5	2.6	573
	30KhHSA	2.99	−1.4	$2\cdot 10^{-6}$	859	–0.59	1.36	20
	FC-16L	1	0	0	0	0	0	0
$HB_{l,i}$	ChNMKh	–0.54	1	2	–50	1	1.7	500
	FMC-11	–0.93	0.83	2.34	546	2.02	2	–233
	30KhHSA	–0.55	1	3.3	0	1	2.5	400
	FC-16L	0.43	1.05	3.5	–250	0	0	0

**Table 3:** Calculated values of some characteristics at each braking cycle.

Characteristic	Brake System	*k* = 1	*k* = 2	*k* = 3	*k* = 4
$f^{\left(k\right)}$	Disc	0.45	0.38	0.32	0.28
	Drum	0.39	0.40	0.41	0.42
$t_{s}{}^{\left(k\right)},\;s$	Disc	1.54	1.73	1.96	2.22
	Drum	6.17	6.00	5.87	5.77
$T_{0}{}^{\left(k\right)},{\;}^{\circ }C$	Disc	20	168	296	418
	Drum	20	53	83	109
$\max T_{m}{}^{\left(k\right)},{\;}^{\circ }C$	Disc	434	542	641	741
	Drum	208	243	272	298
$\max T_{f}{}^{\left(k\right)},{\;}^{\circ }C$	Disc	103	70	53	42
	Drum	237	226	220	216
$\max T_{\max}{}^{\left(k\right)},{\;}^{\circ }C$	Disc	482	560	666	753
	Drum	353	387	416	443

## Data Availability

No new data were created or analyzed in this study. Data sharing is not applicable to this article.

## Nomenclature

$A_{a}$	area of the nominal contact surface ($m^{2}$)
$A_{c}$	area of the contour contact region ($m^{2}$)
$A_{r}$	area of the real contact region ($m^{2}$)
$A_{\mathrm{vent}}$	area of the ventilated surface of the disc or drum ($m^{2}$)
$b_{0}$	parameter of the reference-surface curve (dimensionless)
c	specific heat ($J\;{\mathrm{kg}}^{-1}K^{-1}$)
d	thickness (m)
$d_{r}$	diameter of an average spot of the real contact region (m)
f	coefficient of friction (dimensionless)
h	coefficient of heat transfer ($W\;m^{-2}K^{-1}$)
$h_{\max}$	maximum roughness height on the friction surface of the disc or drum ((m))
HB	Brinell hardness (Pa)
K	thermal conductivity ($W\;m^{-1}K^{-1}$)
n	number of braking in RST brake mode
p	contact pressure (Pa)
$p_{0}$	nominal value of the contact pressure (Pa)
q	specific power of friction ($W\;m^{-2}$)
$q_{0}$	nominal value of the specific power of friction ($W\;m^{-2}$)
$r_{av}$	average rounding radius of roughness on the friction surface ($r_{av}$)
t	time (s)
$t_{b}$	time of performance of all RST mode of braking (s)
$t_{c}$	cooling time at acceleration (s)
$t_{i}$	time of pressure increase (s)
$t_{s}$	time of braking (s)
T	temperature (${}^{\circ }C$)
$T_{0}$	initial (volumetric) temperature (${}^{\circ }C$)
$T_{f}$	flash temperature (${}^{\circ }C$)
$T_{m}$	mean temperature (${}^{\circ }C$)
$T_{\max}$	maximum temperature (${}^{\circ }C$)
V	velocity ($m\;s^{-1}$)
$V_{0}$	initial velocity ($m\;s^{-1}$)
$W_{0}$	initial kinetic energy (J)
z	axial coordinate ((m))
**Greek Symbols**	
ν	parameter of the reference-surface curve (dimensionless)
ρ	density ($\mathrm{kg}\;m^{-3}$)
**Index**	
upper k	number of a stage of braking
lower l	number of the main (l=1) and frictional (l=2) elements of the friction couple

## References

[1] Chichinadze A.V., Eiss N.S. (1984). Polymers in Friction Assembles of Machines and Devices: A Handbook.

[2] Chichinadze A.V. (1995). Processes in heat dynamics and modeling of friction and wear (dry and boundary friction). Tribol. Int..

[3] Day A.J. (2014). Braking of Road Vehicles.

[4] Adamowicz A., Grzes P. (2011). Influence of convective cooling on a disc brake temperature distribution during repetitive braking. Appl. Therm. Eng..

[5] Adamowicz A. (2016). Effect of convective cooling on temperature and thermal stresses in disk during repeated intermittent braking. J. Frict. Wear.

[6] Adamowicz A. (2016). Thermal stressed state of a disk in the process of multiple braking. Mater. Sci..

[7] Kang S.S., Cho S.K. (2012). Thermal deformation and stress analysis of disk brakes by finite element method. J. Mech. Sci. Technol..

[8] Yevtushenko A., Kuciej M., Grzes P., Wasilewski P. (2017). Temperature in the railway disc brake at a repetitive short-term mode of braking. Int. Com. Heat Mass Trans..

[9] Chichinadze A.V., Braun E.D., Ginzburg A.G., Ignat’eva Z.V. (1979). Calculation, Testing and Selection of Friction Couples.

[10] Ginsburg A.G., Romashko A.M., Titarenko V.F. (1974). Calculation of temperature regime of a disc rail brake. Calculation and Modeling of Operation Mode of Breaking and Friction Devices.

[11] Lee K. (1999). Numerical Prediction of Brake Fluid Temperature Rise during Braking and Heat Soaking.

[12] Nosko A.L., Nosko A.P. (2005). Cooling of braking devices of lifting-and-transport machines. Her. Bauman Mosc. State Tech. Univ. Ser. Mech. Eng. Mach. Sci..

[13] Yevtushenko A.A., Grzes P. (2010). The FEM-modeling of the frictional heating phenomenon in the pad/disc tribosystem (a review). Num. Heat Transf. Part A.

[14] Wasilewski P. (2020). Frictional heating in railway brakes: A review of numerical models. Arch. Comput. Methods Eng..

[15] Grzes P. (2019). Maximum temperature of the disc during repeated braking applications. Adv. Mech. Eng..

[16] Yevtushenko A., Kuciej M. (2020). Calculation of friction characteristics of disc brakes used in repetitive short-term braking mode. J. Frict. Wear.

[17] Yevtushenko A.A., Kuciej M. (2012). One-dimensional thermal problem of friction during braking: The history of development and actual state. Int. J. Heat Mass Transf..

[18] Yevtushenko A.A., Kuciej M., Topczewska K. (2017). Analytical model for investigation of the effect of friction power on temperature in the disk brake. Adv. Mech. Eng..

[19] Yevtushenko A.A., Kuciej M., Topczewska K. (2019). Effect of the temporal profile of the friction power on temperature of a pad-disc brake system. J. Theoret. Appl. Mech..

[20] Yevtushenko A.A., Kuciej M., Topczewska K. (2020). Frictional heating during braking of the C/C composite disc. Materials.

[21] Yevtushenko A.A., Grzes P. (2020). Initial selection of disk brake pads material based on the temperature mode. Materials.

[22] Yevtushenko A.A., Grzes P., Adamowicz A. (2020). The temperature mode of the carbon-carbon multi-disc brake in the view of the interrelations of its operating characteristics. Materials.

[23] Chichinadze A.V., Kozhemyakina V.D., Suvorov A.V., Strebezev M.K., Serik A.B. (2007). Temperature field under model test of ring specimens at two side contact on new universal friction machine IM-58-T2. Frict. Lubr. Mach. Mech..

[24] Chichinadze A.V., Kozhemyakina V.D., Suvorov A.V. (2010). Method of temperature-field calculation in model ring specimens during bilateral friction in multidisc aircraft brakes with the IM-58-T2 new multipurpose friction machine. J. Frict. Wear.

[25] Evtushenko O., Kuciej M., Topczewska K. (2020). Determination of the maximal temperature of a pad–disc tribosystem during one-time braking. Mater. Sci..

[26] Chichinadze A.V., Matveevskii R.M., Braun E.P. (1986). Materials in Triboengineering of Unsteady Processes.

[27] Topczewska K. (2018). Influence of the time of increase in contact pressure in the course of braking on the temperature of a pad-disc tribosystem. Mater. Sci..

[28] Demkin N.B., Izmailov V.V., Korotkov M.A. (1976). Estimation of the deformation of rough spheres and cylinders in compression. Wear.

[29] Demkin N.B., Ryzhov E.V. (1981). Surface Quality of Machine Contact Parts.

[30] Grzes P. (2018). Finite element solution of the three-dimensional system of equations of heat dynamics of friction and wear during single braking. Adv. Mech. Eng..

[31] Grzes P. (2017). Determination of the maximum temperature at single braking from the FE solution of heat dynamics of friction and wear system of equations. Num. Heat Transf. Part A.

[32] Yevtushenko A., Kuciej M., Och E., Yevtushenko O. (2016). Effect of the thermal sensitivity in modeling of the frictional heating during braking. Adv. Mech. Eng..

